# Oral sodium butyrate alleviates motor deficits and dopaminergic neuronal loss in 6-hydroxydopamine-induced Parkinson’s disease rat model

**DOI:** 10.1186/s12868-026-01046-x

**Published:** 2026-09-09

**Authors:** Shun-Tai Yang, Yang-Ching Chen, Tzu-Jen Kao, Chi-Wei Kuo, Ching-Lu Chen, Shu-Wei Huang, Shu-Yu Chung, Hsien-Yu Fan, Wan-Ling Tsai

**Affiliations:** 1 Department of Neurosurgery, Shuang Ho Hospital, Taipei Medical University, New Taipei City, 235 Taiwan; 2 Taipei Neuroscience Institute, Taipei Medical University, Taipei, 110 Taiwan; 3 Department of Neurosurgery, School of Medicine, College of Medicine, Taipei Medical University, Taipei, 110 Taiwan; 4 Graduate Institute of Medical Sciences, College of Medicine, Taipei Medical University, Taipei, 110 Taiwan; 5 Department of Family Medicine, School of Medicine, College of Medicine, Taipei Medical University, Taipei, 110 Taiwan; 6 School of Nutrition and Health Sciences, College of Nutrition, Taipei Medical University, Taipei, 110 Taiwan; 7 Department of Family Medicine, Wan Fang Hospital, Taipei Medical University, Taipei, 110 Taiwan; 8 Graduate Institute of Clinical Medicine, College of Medicine, Taipei Medical University, Taipei, 110 Taiwan; 9 Graduate Institute of Metabolism and Obesity Sciences, Taipei Medical University, Taipei, 110 Taiwan; 10 Nutrition Research Center, Taipei Medical University Hospital, Taipei, 110 Taiwan; 11 Ph.D. Program in Medical Neuroscience, College of Medical Science and Technology, Taipei Medical University and National Health Research Institutes, Taipei, 110 Taiwan; 12 International Master Program in Medical Neuroscience, College of Medical Science and Technology, Taipei Medical University, Taipei, 110 Taiwan; 13 School of Physical Therapy, Graduate Institute of Rehabilitation Science, Chang Gung University, Taoyuan, 333 Taiwan; 14 Emergency Department, Wan Fang Hospital, Taipei Medical University, Taipei, 116 Taiwan; 15 Department of Applied Science, National Taitung University, Taitung, 950 Taiwan; 16 Department of Nursing, Asia University, Taichung, 413 Taiwan; 17 School of Nursing, College of Nursing, Fooyin University, Room 631, B Building, No. 151, Jinxue Rd., Daliao Dist., Kaohsiung, 831301 Taiwan

**Keywords:** Short-chain fatty acid, Parkinson’s disease, Neuroprotection, 6-hydroxydopamine, Rat, Mendelian randomization

## Abstract

**Background:**

Short-chain fatty acids regulate intestinal homeostasis and modulate the gut–brain axis, suggesting they may be applicable in adjunctive treatments for Parkinson’s disease (PD). However, the therapeutic efficacy of sodium butyrate (NaB) in PD remains unclear. Using appropriate PD animal models may aid in clarifying the therapeutic effects of NaB and facilitate the development of NaB-based PD treatment strategies. We comprehensively evaluated the therapeutic effects of oral NaB in rats with 6-hydroxydopamine (6-OHDA)-induced PD.

**Methods:**

We induced hemiparkinsonism in rats by administering unilateral 6-OHDA in the medial forebrain bundle. At 24 h after confirming lesion development, some rats were randomized to receive oral NaB (administered daily) over 4 weeks. Behavioral assessments—comprising gait analysis, open-field activity, and apomorphine-induced rotations—were performed longitudinally in weeks 1 and 4. Finally, dopaminergic neuronal degeneration was evaluated to correlate functional recovery with structural neuroprotection.

**Results:**

The 4-week oral NaB intervention significantly alleviated 6-OHDA-induced motor deficits (i.e., locomotor activity, akinesia, and gait) and anxiety-like behavior; however, it did not alleviate apomorphine-induced rotations. Tyrosine hydroxylase immunohistochemical staining confirmed these findings, indicating that the dopaminergic neuron survival rate was significantly higher in the NaB-treated groups than in the sham control group.

**Conclusions:**

Early long-term oral NaB treatment afforded neuroprotection and alleviated motor and non-motor deficits (such as gait and central time in the open field) in rats with 6-OHDA-induced PD. Our model indicates the therapeutic potential of oral NaB and provides a translational framework for investigating the mechanisms of NaB therapy in PD and associated neurological disorders.

## Introduction

Parkinson’s disease (PD) remains a major health challenge, second in prevalence only to Alzheimer’s disease among neurodegenerative disorders globally. It affects 7 to 10 million individuals and approximately 1% of the population aged > 60 years worldwide [1–3]. PD is pathologically characterized by degeneration of dopaminergic neurons within the substantia nigra pars compacta (SNpc), leading to various motor impairments, including tremors, bradykinesia, postural instability, and gait dysfunction [1, 2, 4–6]. Individuals with PD may also present with nonmotor manifestations, including gastrointestinal issues, sleep disorders, depression, and cognitive impairment. The standard pharmacological treatments for PD are dopamine replacement therapy [typically using L-3,4-dihydroxyphenylalanine (levodopa)] or dopamine agonist administration. Although dopaminergic medications can help manage early-stage PD symptoms, their long-term administration typically leads to complications. Within 5–10 years of initiating levodopa treatment, most patients experience side effects such as motor fluctuations, levodopa-induced dyskinesia, postural instability, freezing of gait, and depression [1, 2, 7–10]. Thus, developing nonpharmacological PD treatments is crucial for overcoming the limitations of existing PD interventions.

Research has indicated the pivotal role of the gut–brain axis in PD [11]. This bidirectional communication network integrates the gastrointestinal tract with the central nervous system (CNS) through complex neural, immune, and endocrine pathways [12–16]. Clinically, constipation typically manifests as a prodromal symptom 10–20 years before the emergence of classic motor symptoms, such as tremor or rigidity [17, 18]. Moreover, in patients with PD, constipation promotes proteolytic metabolism over carbohydrate fermentation, thereby causing gut microbiome (GM) dysbiosis [19]. GM dysbiosis then compromises the integrity of the intestinal barrier, and pathogens and toxic substances can enter the bloodstream [20, 21]. The resulting gut–brain axis disruption leads to dysregulated immune responses followed by chronic neuroinflammation [21, 22]. Consequently, neurotoxic misfolded proteins accumulate in and around CNS cells, causing neuronal death [23]. The related evidence confirms the Braak hypothesis: PD pathology, particularly alpha-synuclein misfolding and aggregation, may originate in the enteric nervous system and reach the brainstem and SNpc through retrograde axonal transport along the vagus nerve [21, 22]. A primary driver of this association is GM dysbiosis, characterized by substantial microbial imbalances, which have been increasingly implicated in PD pathogenesis [13, 21].

Studies have explored nonpharmacological PD treatment approaches that focus on GM regulation and short-chain fatty acid (SCFA) enhancement. By restoring the intestinal barrier and reducing systemic inflammation, these approaches are a potential breakthrough for neuroprotection in patients with PD. SCFAs can cross the blood–brain barrier to reach the brain [24, 25]. They can also promote histone deacetylase inhibition and suppress nuclear factor (NF)-κB activation, attenuating proinflammatory cytokine and chemokine production [24]. Moreover, SCFAs can maintain blood–brain barrier homeostasis by upregulating tight junction proteins and can act on neurons and immune cells through G protein-coupled receptors, subsequently downregulating downstream signaling pathways, including MAPK and NF-κB [26, 27]. These microbial metabolites eventually reach the CNS, modulating microglial function and regulating innate immune responses within the brain [21]. Reflecting these multidimensional interactions, at the frontier of biomedical science, the gut–immune–brain axis represents a sophisticated bidirectional axis through which the microbiome orchestrates host immunity, metabolic homeostasis, and neurobiology [21]. Preclinical evidence indicates that sodium butyrate (NaB) alleviates hepatic inflammation, prevents myometrial contraction and spontaneous preterm birth, and suppresses labor onset [17]. Furthermore, oral administration of SCFA-producing fecal microbiota from young donors alleviated behavioral deficits and suppressed cerebral and intestinal inflammation in aged poststroke mice [28]. However, no studies exploring the direct administration of butyrate or NaB to mitigate PD pathology are present in the ClinicalTrials.gov and Cochrane Central Register of Controlled Trials databases, potentially due to real-world limitations. Although SCFA supplementation has been demonstrated to have clinical efficacy in patients with inflammatory bowel disease [17], few studies have investigated its long-term effects in PD animal models. In addition, several critical factors regarding NaB treatment, such as the optimal protocol and underlying mechanisms, remain unclear. Therefore, additional experimental studies are warranted to elucidate the therapeutic value and clinical indications of NaB in PD treatment.

PD animals are considered optimal models for translational assessments of PD pathogenesis. They enable experimentation under stable conditions with standardized treatment protocols and controlled assessment methods, which eliminates experimental discrepancies and clarifies treatment outcomes. However, the long-term effects of NaB on PD-related symptoms and NaB’s potential neuroprotective mechanisms in animal models remain unclear. In the current study, we evaluated the therapeutic potential of NaB in a neurotoxin-induced PD rat model by conducting dopamine depletion analysis and comprehensive behavioral assessments (including detailed time-course analyses of motor symptoms such as gait patterns, open-field locomotor activity, and drug-induced rotation). We hypothesized that long-term NaB intervention mitigates the progression of motor dysfunction and exerts neuroprotective effects on dopaminergic neurons. This study’s insights may have considerable translational relevance to the development of novel therapeutic applications of NaB in PD and other neurodegenerative disorders.

## Material and methods

### Animals

To minimize the potential effects of hormones on PD lesions [29], we used 12 male Sprague Dawley rats (age, 9 weeks; body weight, 300–350 g) purchased from BioLASCO Taiwan (Taipei City, Taiwan). The rats were housed in a temperature-controlled facility (25 °C) under a 12-h light/dark cycle (lights on at 07:00), and food and water were provided ad libitum. All animal procedures were approved by the Institutional Animal Care and Use Committee (IACUC) of Taipei Medical University, and every effort was made to minimize the number of animals used.

### PD rat model

To establish a PD model, we intracerebrally injected rats with the neurotoxin 6-hydroxydopamine (6-OHDA). All surgical procedures and animal preparations were performed in accordance with protocols established previously [1, 30–32]. The rats were anesthetized through an intraperitoneal injection of Zoletil 50 (50 mg/kg; Virbac, Carros, France) and xylazine (10 mg/kg; Rompun; Bayer, Barmen, Germany) and were then mounted on stereotaxic apparatus (Model 940; David Kopf Instruments, Tujunga, CA, USA). A 2-cm midline incision was made along the scalp, and the skull surface was carefully cleared using H_2_O_2_ to expose the bregma. A 10-µL microsyringe (84877; Hamilton, Reno, NV, USA) was employed to inject 6-OHDA solution (8 µg of 6-OHDA dissolved in 4 µL of 0.02% ascorbic saline; Sigma-Aldrich, Burlington, MA, USA) at a rate of 0.5 µL/min into the left medial forebrain bundle (anteroposterior: −4.3 mm; mediolateral: +1.6 mm; dorsoventral: −8.2 mm) after consulting the stereotaxic brain atlas of [33]. The needle was maintained in situ for 5 min and then slowly withdrawn to prevent backflow along the injection tract. After 1 week, the effectiveness of the PD lesion was verified using an apomorphine-induced rotation test. Rats that did not exhibit contralateral rotation were excluded from further analysis.

### Experimental design and intragastric NaB treatment

**Fig. 1:**
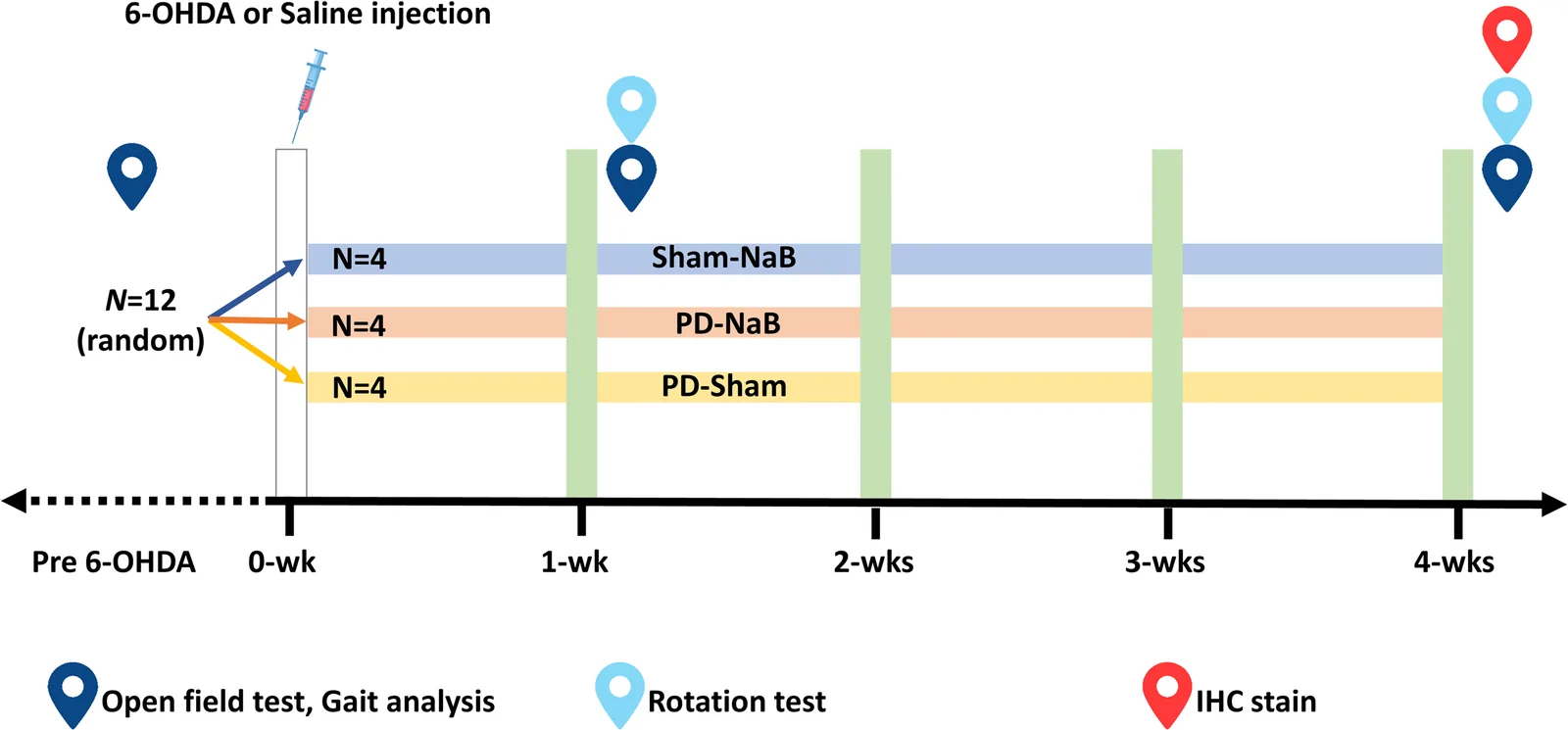
Experimental design for oral NaB treatment. NaB was administered daily over 4 consecutive weeks. Behavioral assessments—the open-field test (for locomotor activity and anxiety-like behavior), gait analysis, and apomorphine-induced rotation test—were conducted at baseline and at 1 and 4 weeks after 6-OHDA-induced lesion development in treated and untreated rats. At 4 weeks after lesion development, IHC staining was performed to evaluate the neuroprotective effects of NaB on dopaminergic neurons and fibers

After their behavioral was trained, the rats were randomly divided into three groups (*n* = 4 per group): Saline sham-NaB, PD-Sham, and PD-NaB groups. The Sham-NaB and PD-NaB groups were administered NaB (50 mg/kg/day) through oral gavage, whereas the PD-Sham group received sterile phosphate-buffered saline (PBS; 100 µL/day; [34]. Sodium butyrate was purchased from Sigma-Aldrich (St. Louis, MO, USA). We previously noted that a high degree of dopamine depletion may limit motor plasticity, preventing motor performance gains [35]. We administered NaB as an early intervention following symptom onset to investigate its therapeutic potential **(**Fig. 1**)**. Treatment was initiated 24 h after lesion development; the treatment groups received NaB through oral gavage once daily over 28 consecutive days. Body weight was recorded weekly. Locomotor activity and anxiety-like behavior were assessed in treatment weeks 1 and 4 by using the rotation test, gait analysis, and the open-field test. A trained, blinded examiner performed all behavioral examinations conducted before and after treatment. Table 1 summarizes the timeline of our behavioral and biochemical analyses. The rats were allowed ≥ 3 h of rest between each test. Once all behavioral assessments in treatment week 4 were complete, we euthanized all rats and collected biological samples for analysis.

**Table 1:** Experimental timeline for behavioral and biochemical assessments of Sham-NaB, PD-Sham, and PD-NaB group rats

Assay	Weeks after 6-OHDA lesion					
	Pre 6-OHDA	0-wk	1-wk	2-wk	3-wk	4-wk
Open-field test	+	-	+	-	-	+
Gait analysis	+	-	+	-	-	+
Rotation test	-	-	+	-	-	+
IHC stain	-	-	-	-	-	+

Each group comprised four male rats

### Behavioral assessment

We performed behavioral assessments on the 6-OHDA-lesioned rats treated or not treated with NaB at baseline and at 1 and 4 weeks after lesion development. These assessments comprised the apomorphine-induced rotation test, open-field test, and gait analysis.

#### Apomorphine-induced rotation test

To determine the rats’ dopamine depletion level after unilateral 6-OHDA infusion, the reliable and conventional apomorphine-induced contralateral rotation test was performed [30, 36–39]. In brief, we injected the rats subcutaneously with apomorphine (0.5 mg/kg, dissolved in 0.02% ascorbic saline; Sigma-Aldrich) and placed them in a 40-cm-diameter bowl. Contralateral (i.e., clockwise) rotations were video-recorded for 60 min and subsequently quantified by a blinded, trained examiner. In general, a rotational response of > 300 turns per hour indicates approximately 90% dopaminergic neuronal loss in the SNpc [30, 40]. This test was also performed 4 weeks after lesion development to confirm the 6-OHDA infusion efficacy. Rats that failed to exhibit contralateral rotation were excluded from further analysis.

#### Open-field test

The open-field test was employed to assess general locomotor activity and anxiety-like behavior in our 6-OHDA-induced PD rats [1, 41, 42]. A digital camera (C930e; Logitech, Newark, CA, USA) was used to monitor the rats when they were placed individually in a black plexiglass arena (100 cm × 100 cm × 40 cm) in accordance with protocols described previously [1, 35, 43]. The total distance the rats traveled and the time they spent immobile over the 10-min testing period were determined using a video-tracking system (Smart v3.0; Panlab SL, Barcelona, Spain). To ensure consistency and eliminate olfactory cues, we thoroughly cleaned the testing arena with 75% ethanol between sessions.

#### Gait analysis

We performed gait analysis to assess motor disturbances in the PD rats before and after NaB treatment. This analysis followed previously described methods [2, 30, 32, 35, 43]. The apparatus comprised a transparent Plexiglas walkway (80 cm × 9 cm × 12 cm) with a 45° tilted mirror positioned beneath it. A high-speed camera (Lumix G9II; Panasonic, Japan) was used to capture sagittal and reflected-downward views at 60 fps. The captured images were processed semiautomatically by using the software program Motion Gait to identify sequential footprints. Finally, spatial parameters (step length, stride length, support base, and foot angle) and temporal parameters (walking speed, stance phase time, swing phase time, and stance/swing ratio) were determined [30, 32, 44].

### Immunohistochemical staining

Dopaminergic neurodegeneration in the nigrostriatal system was assessed using tyrosine hydroxylase (TH) immunohistochemical (IHC) staining [1, 30, 45]. After behavioral testing at 4 weeks post-lesion, rats with 6-OHDA-induced PD from the experimental and sham groups were sacrificed. The animals were deeply anesthetized with 5% isoflurane and transcardially perfused with normal saline followed by 4% paraformaldehyde (PFA) in 0.1 M PBS. Thereafter, the rat brains were harvested and postfixed in 4% PFA at 4 °C overnight. For cryoprotection, brain tissue was dehydrated in 30% sucrose in PBS at 4 °C for 5 days and then stored at − 81 °C. Sagittal sections (thickness, 30 μm) were prepared in a cryostat (CM1950; Leica) at − 21 °C. IHC staining and quantitative analyses were performed using protocols established previously [1, 2]. The primary antibodies used were rabbit anti-TH (1:1000; AB152; Millipore, Burlington, MA, USA).

### Data analysis

Statistical analyses were conducted using GraphPad Prism version 11.0 (GraphPad Software, La Jolla, CA, USA). Data are presented as means ± standard errors of the means (SEMs). Behavioral outcomes measured over time were analyzed using two-way repeated-measures analysis of variance (ANOVA), with group (NaB vs. sham) as the between-subjects factor and time as the within-subjects factor. When a significant main effect or interaction was identified, post hoc analysis was performed using Bonferroni correction. Comparisons of two independent groups at specific time points were performed using the independent-samples *t* test. For all tests, statistical significance was set at *p* < 0.05.

## Results

### Effect of NaB on body weight

**Fig. 2:**
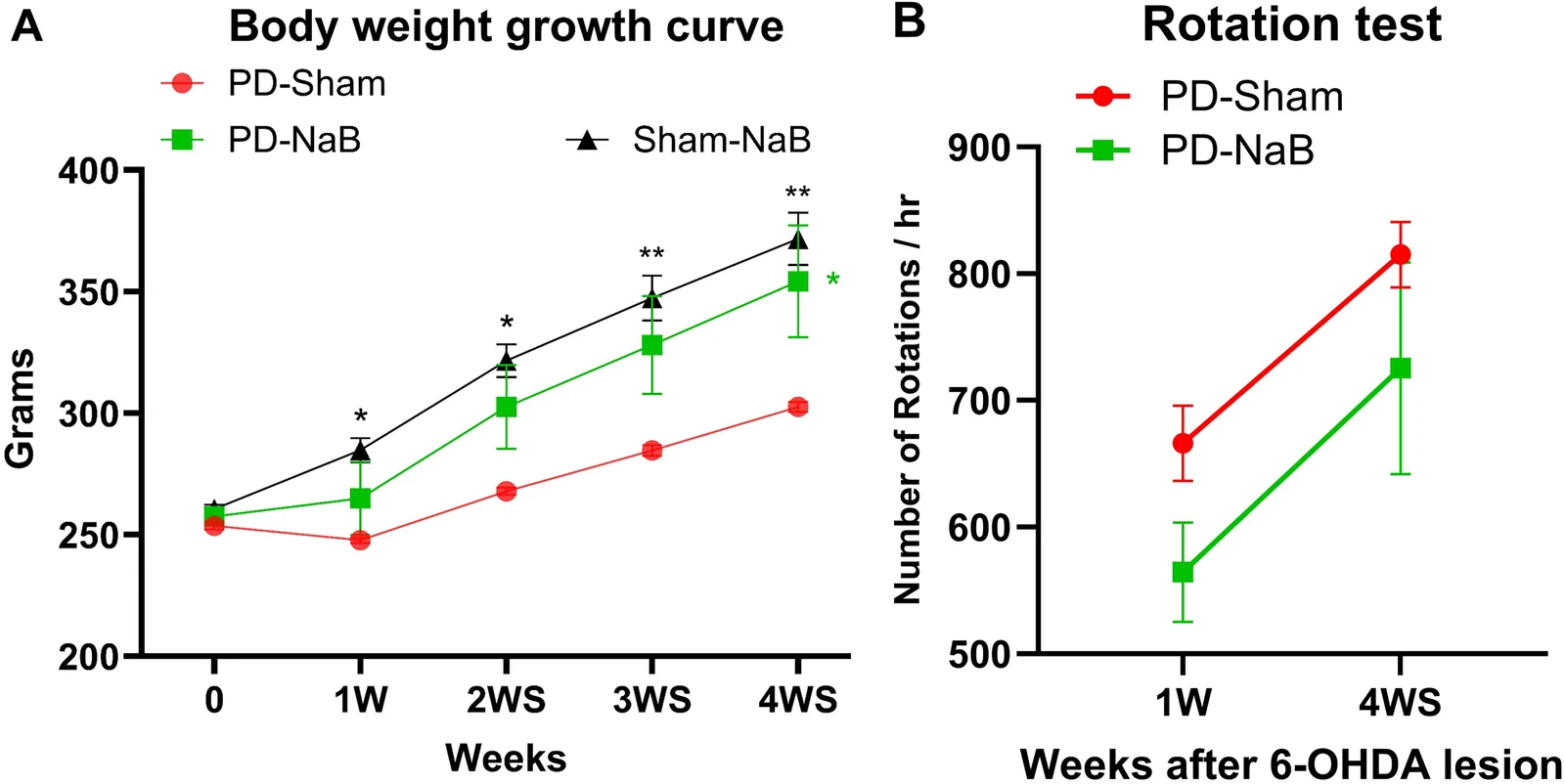
Physiological and behavioral assessments of PD rats. **(A)** Growth curve based on body weight. **(B)** Evaluation of nigrostriatal lesion development using the rotation test. Data are expressed as means ± SEMs. *p < 0.05, **p < 0.01, ***p < 0.001, and ****p < 0.0001, indicating significant between-group differences

To investigate the effect of NaB on body weight, we weighed the rats in postnatal weeks 8 and 12. In postnatal week 8, the body weights of the PD-Sham and PD-NaB groups did not differ significantly **(**Fig. 2A**)**. However, by postnatal week 12, the body weight was significantly lower in the PD-Sham group than in the PD-NaB group. Additionally, weight gain was lower in the PD-Sham group than in the PD-NaB group **(**Table 2**)**.

**Table 2:** Basic characteristics of PD-Sham, PD- NaB, and Sham-NaB group rats

Characteristics	Sham-NaB	PD-Sham	PD-NaB
Initial weight [g]	260.50 ± 1.85	253.67 ± 1.50	257.50 ± 3.75
1-wk [g]	284.75 ± 4.96*	247.67 ± 1.28	265.00 ± 15.03
2-wk [g]	321.50 ± 6.74*	267.83 ± 1.58	302.50 ± 17.27
3-wk [g]	347.25 ± 9.29**	284.67 ± 2.28	328.00 ± 20.15
Final weight [g]	371.75 ± 10.75**	302.50 ± 2.17	354.25 ± 23.06*
Weight gain [g]	111.25 ± 10.91	48.83 ± 2.64	96.75 ± 23.37

Initial and final weights were measured at postnatal weeks 8 and 12, respectively

Each group comprised four male rats

Data are presented as means ± SEMs

*p < 0.05, **p < 0.01, ***p < 0.001, ****p < 0.0001, versus control group

### Effect of NaB on apomorphine-induced rotation

To evaluate the progression of motor impairment in PD rats, we performed apomorphine-induced rotation tests at 1 and 4 weeks post-lesion. By week 4, all rats with lesions exhibited robust contralateral rotation, and eight rats met the inclusion criteria enabling further analysis. These animals were distributed equally between the PD-Sham (*n* = 4) and PD-NaB (*n* = 4) groups. Repeated-measures ANOVA revealed significant differences in the main effects of time (*F*_(1, 4)_ = 7.394, *p* = 0.0530), group (*F*_(1, 6)_ = 9.020, *p* = 0.0239), and the time × group interaction (*F*_(1, 4)_ = 0.008152, *p* = 0.9324). Moreover, Šídák multiple comparison test revealed that at each time point, the difference was mainly influenced by the treatment effects of NaB, mostly observed in terms of the number of rotations per hour in weeks 1 (*p* = 0.2219) and 4 (*p* = 0.2545) after lesion development **(**Fig. 2B**)**.

### Effects of NaB on anxiety-like behavior and locomotor activity in open-field test

The open-field test was used to assess locomotor activity, with total distance traveled and immobility time employed as the primary metrics. These parameters were used to assess differences in general motor function between the PD-Sham and PD-NaB groups from the prelesion stage through to 1 and 4 weeks after lesion development.

**Fig. 3:**
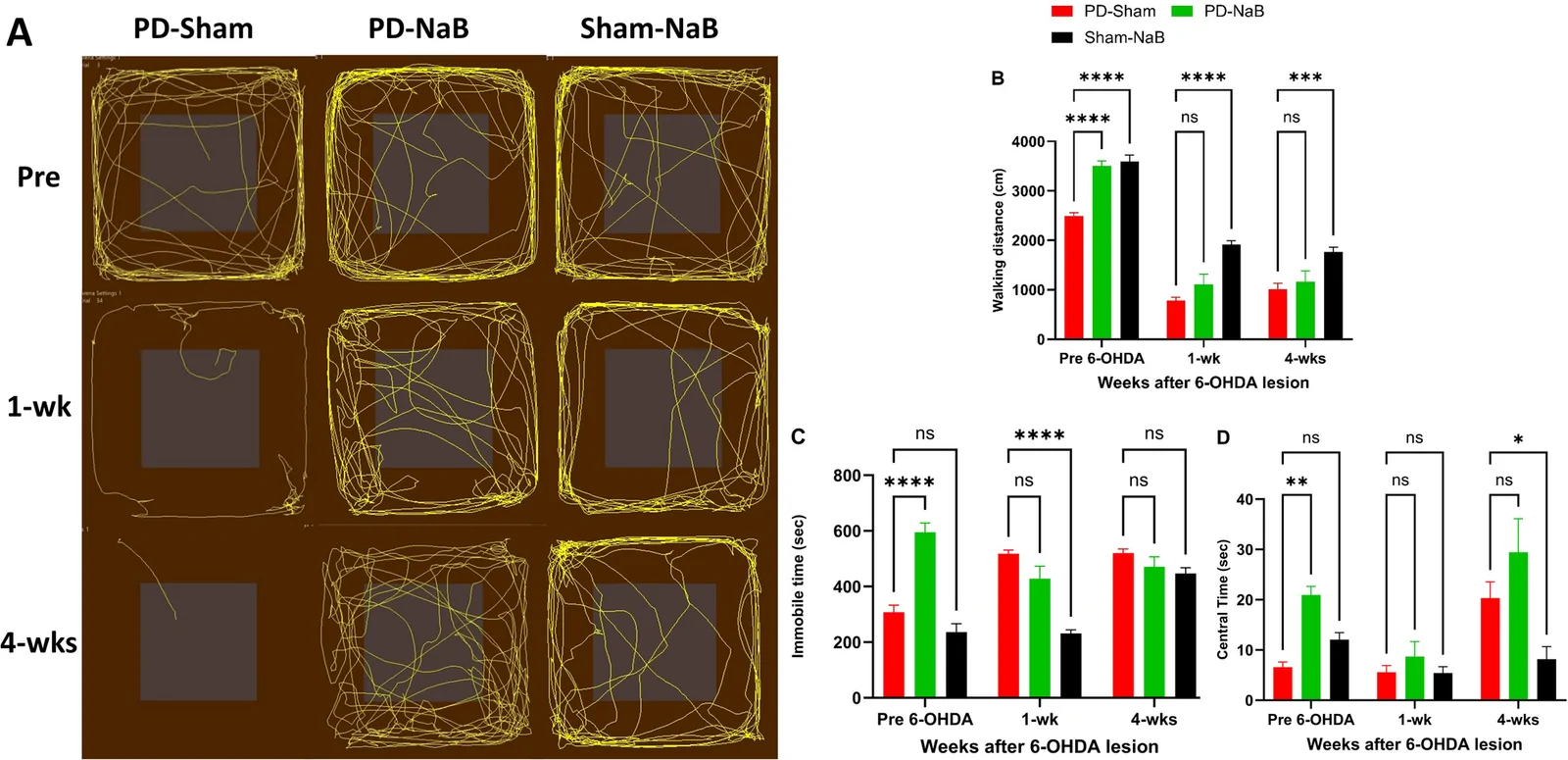
**(A)** Time-course analysis of anxiety-like behavior and locomotor activity in the open-field test before and after 6-OHDA-induced lesion development. **(B–D)** Significant differences in walking distance (**B**), immobility time (**C**), and time spent in the center (**D**) between the PD-Sham, PD-NaB, and Sham-NaB groups after lesion development. Data are expressed as means ± SEMs. *p < 0.05, **p < 0.01, ***p < 0.001, and ****p < 0.0001, indicating significant between-group differences

We compared the total walking distance traveled by the PD-NaB and PD-Sham rats after lesion development **(**Fig. 3A**).** Repeated-measures ANOVA revealed significant differences in main effects of time (*F*_(2, 27)_ = 190.4, *p* < 0.0001), group (*F*_(2, 27)_ = 39.09, *p* < 0.0001), and the time × group interaction (*F*_(4, 27)_ = 3.347, *p* = 0.0238). Moreover, the results of post hoc analysis performed using Dunnett’s test indicated that NaB significantly influenced the total distance traveled at all time points, most notably when the Sham-NaB and PD-Sham groups were compared. At 1 week after lesion development, this test revealed the following results: PD-Sham vs. PD-NaB (*p* = 0.1979) and PD-Sham vs. Sham-NaB (*p* < 0.0001). At 4 weeks after lesion development, the results were as follows: PD-Sham vs. PD-NaB (*p* = 0.6404) and PD-Sham vs. Sham-NaB (*p* = 0.0012; Fig. 3B).

Regarding immobility time, repeated-measures ANOVA revealed significant differences in the main effects of time (*F*_(2, 27)_ = 11.58, *p* = 0.0002), group (*F*_(2, 27)_ = 39.47, *p* < 0.0001), and the time × group interaction (*F*_(4, 27)_ = 18.14, *p* < 0.0001). Furthermore, post hoc analysis conducted using Dunnett’s test indicated that the observed differences were primarily driven by NaB treatment across all time points, with the most significant effects being noted at 1 week after lesion development. At this time point, the test revealed the following results: PD-Sham vs. PD-NaB (*p* = 0.0559) and PD-Sham vs. Sham-NaB (*p* < 0.0001). At 4 weeks after lesion development, the results were as follows: PD-Sham vs. PD-NaB (*p* = 0.3590) and PD-Sham vs. Sham-NaB (*p* = 0.1286; Fig. 3C**)**.

Regarding time spent in the center of the open field, repeated-measures ANOVA revealed significant differences in the main effects of time (*F*_(2, 27)_ = 13.80, *p* < 0.0001), group (*F*_(2, 27)_ = 11.72, *p* = 0.0002), and the time × group interaction (*F*_(4, 27)_ = 3.723, *p* = 0.0154). Dunnett’s test revealed that for all time points, NaB treatment was the primary driver of the variation in time spent in the center, with its impact being the most significant at 4 weeks after lesion development. At 1 week after lesion development, this test yielded the following results: PD-Sham vs. PD-NaB (*p* = 0.6881) and PD-Sham vs. Sham-NaB (*p* < 0.9988). At 4 weeks after lesion development, the results were as follows: PD-Sham vs. PD-NaB (*p* = 0.0712), and PD-Sham vs. Sham-NaB (*p* = 0.0142; Fig. 3D**)**.

### Effect of NaB on gait pattern

**Fig. 4:**
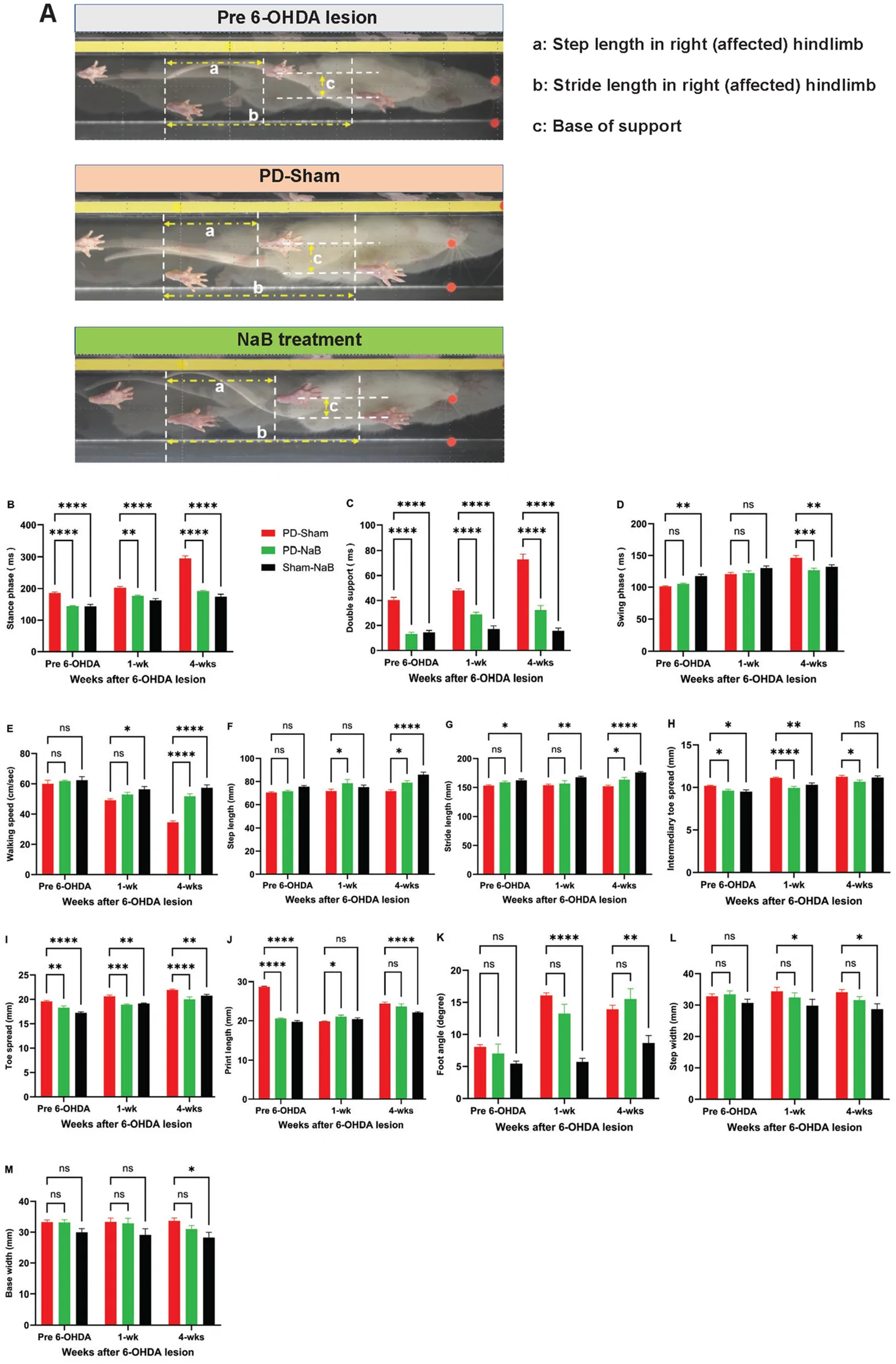
Comparison of stepping patterns at baseline (prelesion) and 4 weeks after lesion development in sham- and NaB-treated PD rats. **(A)** Sequential footprint records showing gait patterns during walkway movement in sham- and NaB-treated rats. **(B–M)** Time-course analysis of stance phase time (**B**), double support time (**C**), swing phase time (**D**), walking speed (**E**), step length (**F**), stride length (**G**), intermediary toe spread (**H**), toe spread (**I**), print length (**J**), foot angle (**K**), step width (L), and base width (**M**) in sham- and NaB-treated PD rats over 4 weeks. NaB mitigated PD-induced reductions in walking speed, step length, and stride length. The NaB groups exhibited a smaller increase in support base than did the PD-Sham group, indicating preserved gait stability. Data are expressed as means ± SEMs. *p < 0.05, **p < 0.01, ***p < 0.001, and ****p < 0.0001, indicating significant between-group differences

We next quantitatively analyzed the effects of NaB treatment on the gait patterns of the PD rats. Figure 4A presents typical footprint images of the PD rats in the sham and NaB treatment groups at baseline (prelesion) and 4 weeks after lesion development. Repeated-measures ANOVA on gait parameters revealed a significant time × group interaction for stance phase (*F*_(4, 27)_ = 20.00, *p* < 0.0001), double support (*F*_(4, 27)_ = 10.88, *p* < 0.0001), swing phase (*F*_(4, 27)_ = 8.412, *p* = 0.0002), walking speed (*F*_(4, 27)_ = 11.22, *p* < 0.0001), step length (*F*_(4, 27)_ = 3.978, *p* = 0.0115), stride length (*F*_(4, 27)_ = 2.290, *p* = 0.0857), intermediary toe spread (*F*_(4, 27)_ = 2.105, *p* = 0.1079), toe spread (*F*_(4, 27)_ = 3.166, *p* = 0.0295), print length (*F*_(4, 27)_ = 58.67, *p* < 0.0001), and foot angle (*F*_(4, 27)_ = 4.998, *p* = 0.0038); these results suggested less gait pattern impairment in the NaB treatment groups than in the PD-Sham group (Fig. 4B–K). No significant group × time interaction was found for step width (*F*_(4, 27)_ = 0.5267, *p* = 0.7170) or base width (*F*_(4, 27)_ = 0.3062, *p* = 0.8712; Fig. 4L and M). Post hoc analysis performed using Dunnett’s test revealed significant differences in stance phase, double support time, step length, intermediary toe spread, and toe spread between 1 and 4 weeks after lesion development (Fig. 4B, C, F, H, and I).

### Quantification of dopaminergic neuronal degeneration

**Fig. 5:**
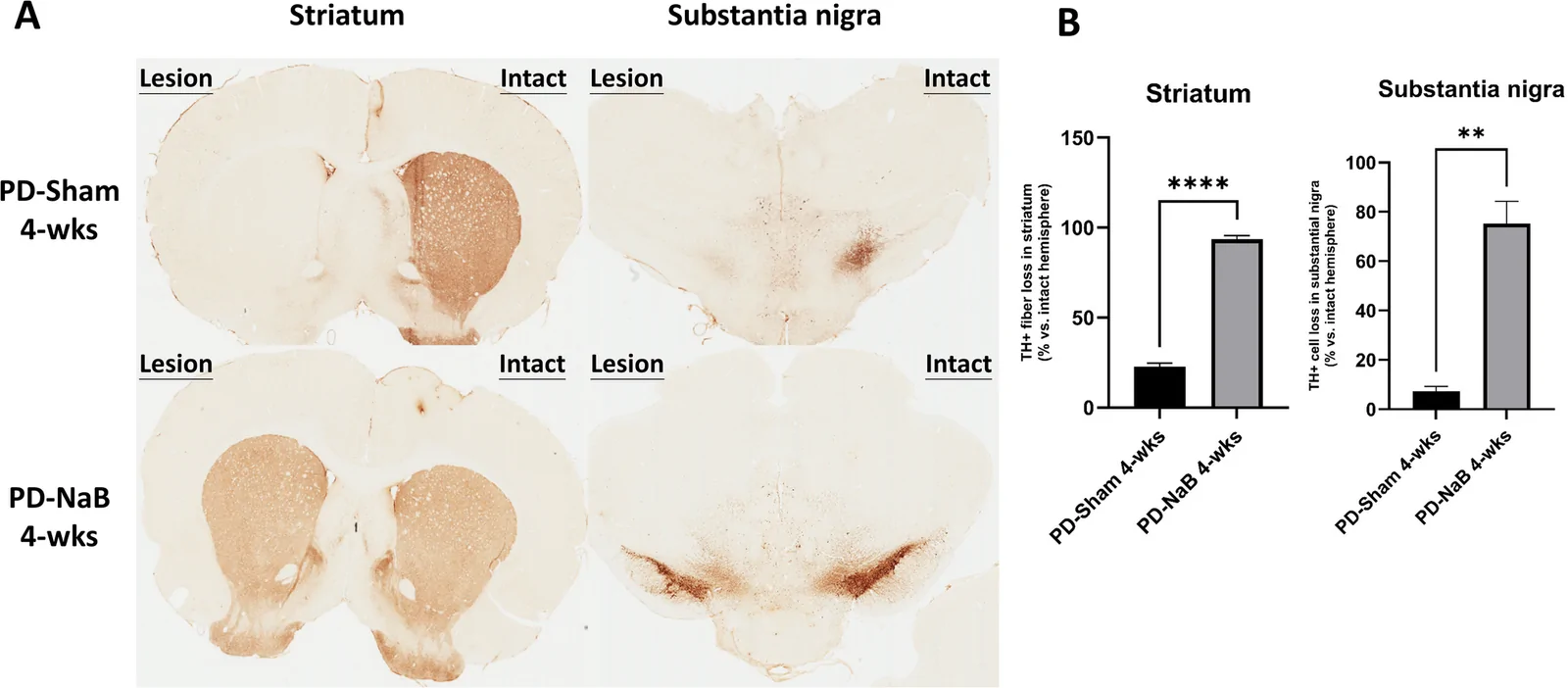
**(A)** Representative images of TH-positive fibers in the striatum and TH-positive neurons in the SNpc in PD-Sham and PD-NaB group rats at 4 weeks after lesion development. **(B)** Quantification and comparison of average TH-positive fiber and neuron density in the PD-Sham and PD-NaB groups

To evaluate the effects of the 4-week NaB treatment on dopaminergic neurons and fibers within the nigrostriatal pathway, we quantified TH-positive cells in the SNpc and TH-positive fibers in the striatum by performing IHC staining. Figure 5A displays representative staining results for the SNpc and striatum in PD rats treated and not treated with NaB. At 4 weeks after lesion development, we noted the loss of both TH-positive fibers in the striatum and TH-positive cells in the SNpc **(**Fig. 5B**)**. Nevertheless, compared with the PD-Sham group, the NaB-treated PD rats exhibited significantly greater preservation of TH-positive fibers in the striatum (Welch’s *t* test; *df* (5.831) = 21.66, *p* < 0.0001) and TH-positive cells in the SNpc (*df* (3.436) = 7.197, *p* = 0.0035).

## Discussion

Recent multi-cohort studies demonstrate that PD is driven by gut-immune-brain cross-talk linked to a distinct gut dysbiosis pattern, characterized by the depletion of SCFA-producing genera, enrichment of opportunistic pathobionts, and functional alterations in gene expression that fuel systemic inflammation and neurodegeneration [18, 21]. Consequently, despite their distinct primary etiologies, PD and depression frequently co-occur. In both clinical cohorts and animal models, non-motor symptoms—such as anxiety, depression, and cognitive decline—often manifest during the prodromal phase, long before classic motor deficits like tremor emerge [35, 43, 46]. This suggests that early gut-induced neuropathological changes may serve as a pivotal driver of subsequent psychological and cognitive dysfunction.

In the current study, we evaluated the therapeutic efficacy of oral NaB in rats with 6-OHDA-induced PD over 4 weeks by comprehensively and quantitatively assessing the motor functions typically impaired in PD. We noted that 4-week NaB treatment effectively mitigated 6-OHDA-induced neurotoxicity, significantly ameliorating both motor and affective behavioral deficits potentially through neuroprotective effects on dopaminergic neurons and fibers. Furthermore, IHC staining revealed that oral NaB significantly preserved dopaminergic neurons and striatal fibers. To the best of our knowledge, this study is the first to comprehensively evaluate the therapeutic effects of oral NaB on multiple motor function domains in a PD rat model. Our findings contribute crucial evidence to the growing body of basic and clinical research exploring long-term NaB treatment as a potential therapeutic strategy for PD.

Several PD animal models have been established, including models in which PD is induced using the neurotoxin 6-OHDA. However, the precise mechanisms underlying 6-OHDA-induced depressive behaviors and cognitive deficits remain unclear. Clinical evidence indicates that patients with PD exhibit both learning and affective impairments, and the nigrostriatal and mesocortical pathways are strongly implicated in anxiety deficits [4]. The open-field test is widely used to assess anxiety-like behavior in PD rat models [35, 43, 46, 47]. In the current study, oral NaB significantly alleviated anxiety-like behavior and locomotor impairments induced by 6-OHDA. Although the between-group differences in the apomorphine-induced rotation test were nonsignificant, a trend toward decreased rotation was noted in the PD-NaB group, suggesting that NaB may provide modest neuroprotection against neurotoxic insults. Oral NaB may exert therapeutic effects by acting as a histone deacetylase inhibitor, elevating striatal levels of neurotrophic factors [e.g., brain-derived neurotrophic factor (BDNF)] and anti-inflammatory mediators [24]. Furthermore, NaB suppresses MAPK and NF-κB activation [48, 49], leading to attenuation of proinflammatory cytokines and chemokines and thereby mitigating 6-OHDA-induced neurotoxicity. SCFAs have been widely reported to upregulate the expression of BDNF, a crucial regulator of neuronal survival, differentiation, and synaptic plasticity [24]. IHC staining indicated that NaB attenuated TH-positive fiber and cell degeneration to some extent in the NaB group. Furthermore, the PD rats subjected to NaB treatment over 4 weeks exhibited significant alleviation of anxiety-freezing-like behavior (immobility time) and gait impairments (walking speed) in the open field test. These findings corroborate clinical evidence indicating that SCFAs can improve cognitive and motor functions in patients with advanced PD [17]. Open-field tests successfully captured NaB-mediated improvements in locomotion and anxiety, reflecting broader systemic recovery (e.g., anti-inflammation and metabolic modulation). However, a high apomorphine dose (0.5 mg/kg) saturated postsynaptic dopamine receptors, creating a ceiling effect that masked treatment differences in rotational behavior [50].

Gait impairments are prevalent among individuals with PD due to dopaminergic neuron degeneration in the SNpc [4, 51]. In our study, we did not quantify gait sequence parameters because we only evaluated the hind limbs. However, studies have indicated that unilateral 6-OHDA lesions in rats alter their gait sequence preferences. The current results demonstrated that gait impairments may not be limited to alterations in walking speed and may manifest in various spatiotemporal and kinematic dimensions. In particular, stride length, step length, double support time, stance phase time, swing phase time, paw angle, toe spread, and intermediate toe spread were significantly different on the lesioned side compared with on the contralateral side; moreover, walking speed differed significantly between the treatment and PD-Sham groups. These results confirm that muscle movement coordination is involved in rat gait behavior. Moreover, behavioral and gait analyses revealed that NaB attenuated the gait deficits of our PD rats. In particular, dopamine deficiency induced significant changes in walking speed, step length, stride length, double support time, stance phase time, swing phase time, foot angle, toe spread, and intermediary toe spread at 1–4 weeks after lesion development. After they were unilaterally injected with 6-OHDA, the rats’ gait performance was found to be bilaterally impaired from 1 week after lesion development, with more severe deficits on the affected side than on the intact side. This observation indicated that gait impairments on one side interact with those on the other. Importantly, we noted that NaB can significantly ameliorate 6-OHDA-induced gait impairments bilaterally.

Nevertheless, several limitations of this study should be acknowledged. Although the sample size (*n* = 4 per group) was minimized in accordance with animal welfare and ethical standards, the consistency between parametric and nonparametric analyses underscores the reliability of our findings. Furthermore, since standard neuronal markers (e.g., NeuN) were not co-labeled with TH in our immunohistochemical analyses, we cannot definitively rule out whether NaB directly prevents neurodegeneration or merely restores TH phenotypic expression in dysfunctional but viable neurons. Future studies employing pan-neuronal markers are warranted to fully elucidate these underlying mechanisms. Overall, our findings provide valuable preliminary evidence supporting the neuroprotective potential of NaB.

## Conclusion

In this study, we delineated progressive changes in PD-related symptoms after oral NaB treatment. In particular, we noted that NaB ameliorates motor dysfunctions and has neuroprotective effects within the dopaminergic system of rats with 6-OHDA-induced PD. Our PD rat model may bridge the gap between animal and human studies into long-term oral NaB treatment. Future studies should focus on clarifying the mechanisms underlying NaB’s therapeutic efficacy, which would enabled the development of optimized oral NaB protocols for treating PD and other neurological diseases in humans.

## Data Availability

All data supporting the findings of this study are available within the paper and its Supplementary Information.

## Abbreviations

SCFA: Short chain fatty acid
PD: Parkinson’s disease
6-OHDA: 6-Hydroxydopamine
TH: Tyrosine hydroxylase
SNpc: Substantia nigra pars compacta
CNS: Central nervous system
GM: Gut microbiota
NaB: Sodium butyrate
IHC: Immunohistochemical
SEM: Standard error of the mean
BDNF: Brain-derived neurotrophic factor
PBS: Phosphate-buffered saline
